# Optimization of the Dose Rate Effect in Tetrazolium Gellan Gel Dosimeters

**DOI:** 10.3390/gels9040334

**Published:** 2023-04-14

**Authors:** Kalin I. Penev, Matt Mulligan, Kibret Mequanint

**Affiliations:** 1 Department of Chemical and Biochemical Engineering, The University of Western Ontario, London, ON N6A 5B9, Canada; 2 Modus Medical Devices Inc., London, ON N6H 5L6, Canada; 3 London Regional Cancer Program, London, ON N6A 5W9, Canada; matt.mulligan@lhsc.on.ca; 4 Biomedical Engineering Graduate Program, The University of Western Ontario, London, ON N6A 5B9, Canada

**Keywords:** gel dosimeters, formulation optimization, dose rate effect, tetrazolium salt dosimeters, ClearView 3D dosimeter

## Abstract

Tetrazolium salts provide an appealing candidate for 3D gel dosimeters as they exhibit a low intrinsic color, no signal diffusion and excellent chemical stability. However, a previously developed commercial product (the ClearView 3D Dosimeter) based on a tetrazolium salt dispersed within a gellan gum matrix presented a noticeable dose rate effect. The goal of this study was to find out whether ClearView could be reformulated in order to minimize the dose rate effect by optimizing of the tetrazolium salt and gellan gum concentrations and by the addition a thickening agent, ionic crosslinkers, and radical scavengers. To that goal, a multifactorial design of experiments (DOE) was conducted in small-volume samples (4-mL cuvettes). It showed that the dose rate could be effectively minimized without sacrificing the integrity, chemical stability, or dose sensitivity of the dosimeter. The results from the DOE were used to prepare candidate formulations for larger-scale testing in 1-L samples to allow for fine-tuning the dosimeter formulation and conducting more detailed studies. Finally, an optimized formulation was scaled-up to a clinically relevant volume of 2.7 L and tested against a simulated arc treatment delivery with three spherical targets (diameter 3.0 cm), requiring different doses and dose rates. The results showed excellent geometric and dosimetric registration, with a gamma passing rate (at 10% minimum dose threshold) of 99.3% for dose difference and distance to agreement criteria of 3%/2 mm, compared to 95.7% in the previous formulation. This difference may be of clinical importance, as the new formulation may allow the quality assurance of complex treatment plans, relying on a variety of doses and dose rates; thus, expanding the potential practical application of the dosimeter.

## 1. Introduction

About half of all cancer patients undergo radiation therapy, making it one of the most common treatment modalities for the disease. However, given the radiation toxicity to healthy tissues and the potential for unwanted side effects, it is important to ensure that the healthy tissue is spared and that most of the radiation dose is delivered to the tumor. To that end, modern radiation therapy relies on personalized radiation treatment plans adjusted to the patient’s anatomy. One way to ensure that the treatment plan and delivery truly conform to the complexity of anatomical features is to perform three-dimensional (3D) dosimetry [1,2]. This can be achieved by dispersing a radiation-sensitive material into a continuous medium and irradiating the volume, thus creating an image of the absorbed dose [2]. Different embodiments of this idea exist, using plastic (e.g., polyurethane), elastic (e.g., silicone), or hydrogel-based continuous media and various signal-generating compounds, such as radiation-sensitive dyes and cross-linkable monomers [3].

Gel dosimeters are particularly appealing due to their high water content, making them nearly water equivalent from a radiological point of view [4]. The two types of gel dosimeters are polymer gels and radiochromic gels [5]. In polymer gel dosimeters, acrylic monomers are first dispersed within a hydrogel matrix (such as gelatin). Radiation-induced polymerization reactions create a 3D image of the absorbed dose [6]. In radiochromic gel dosimeters, the signal is generated by a radiochemical color change of small molecules (dyes or indicators), dispersed within the gel matrix [3]. The preparation and utilization of polymer gel dosimeters is an intensive multiday process that requires adhering to a strict protocol, including a necessary waiting period of 24 to 46 h between irradiation and scanning [7], which arguably makes polymer 3D gel dosimetry both logistically challenging and expensive, as one has to account for material, labor and magnetic resonance imaging (MRI) costs. By contrast, in radiochromic gel dosimeters the chemical reaction typically takes less than an hour to complete, and scanning is performed on dedicated and relatively low-cost optical computed tomography scanners [8,9].

Tetrazolium salts are a particularly appealing radiochromic indicator, as they are typically colorless and very water soluble owing to their charged nature, but upon radiochemical reduction convert into intensely colored and water insoluble formazan dyes, creating a permanent optical image of the absorbed dose [10]. 3D dosimetry based on the reduction of tetrazolium salts has been achieved with different gel-forming materials, including gelatin and gellan gum [11,12], as well as Pluronic F-127 [13]. In all cases, the dosimeters showed low initial optical background before irradiation, lack of signal diffusion after irradiation, and multiday stability before and after irradiation. However, as initially developed, tetrazolium salt gel dosimeters tend to have lower sensitivity compared to other radiochromic 3D dosimeters [3], as well as an appreciable dose rate effect at dose rates below approximately 600 cGy/min [12]. Developed in our laboratory, a new gel dosimeter has been commercialized under the name ClearView by Modus Medical Devices Inc. (Modus QA), (London, ON, Canada) and primarily used for geometric registration of small-beam irradiation [14,15,16] and end-to-end dosimetric evaluation of single isocenter multi-target treatment and prostate plan validation [17]. The initial results indicated that the relatively low sensitivity to dose and the dose rate effect of the sensitivity may cause misregistration of the delivered dose plan. To address this issue, we showed that the addition of radical scavengers, such as methyl paraben and lithium formate [18] and/or use of novel tetrazolium salts [19] may improve the sensitivity of the dosimeter. However, these results were achieved at the cost of higher initial optical background and/or decreased chemical and structural stability of the gel dosimeters. In our latest communication [20], we showed an optimization on all main dosimetric parameters by using small-volume dosimeters (4 mL cuvettes). For practical application, however, the results from cuvette studies may not translate directly to the properties of the dosimeter in the required larger volume samples. Therefore, formulation optimization still needed to be performed in anatomically relevant volumes using realistic irradiation plans, which is the objective of this paper. Herein is a multifactorial design of experiment, aimed at minimizing the dose rate dependence in large-volume dosimeters.

In principle, decreasing the dose rate effect could be achieved by decreasing the gellan gum concentration or by increasing the concentration of the tetrazolium salt [18,20]. Radical scavengers (such as alcohols) are also required to be present in the gel for the radiolysis of tetrazolium salts to achieve a high yield of the formazan dye [21]. Further, the gel dosimeter needs to be optically clear, dimensionally stable, and chemically stable during storage and handling. To address all these requirements, several improvements in the experimental design have been made here relative to the prior communications. First, to improve the handling of gels with low total solids loading, the previously selected thickening agent, polyacrylamide (PAM), with a molecular weight of 250 kDa, was substituted here by PAM with an order of magnitude higher molecular weight (5–6 MDa). Based on preliminary tests, this would allow similar gel handling at a much lower PAM loading. Second, the lithium formate, previously used as both a crosslinking agent and a radical scavenger caused a drastically increased rate of auto-reduction [18], likely due to its alkaline reaction and the decreased stability of tetrazolium salts at elevated pH [21]. Here, lithium formate was substituted by the similar radical scavenger lithium lactate, buffered with lactic acid to pH 5.3, which is the native pH for gellan gum [22]. The same effect could not be achieved with a lithium formate—formic acid buffer, due to the volatility of the latter. The goal was to test whether increased sensitivity can be obtained without decreased chemical stability of the dosimeter. Alternatively, lithium chloride was used as a pH-neutral crosslinking agent without radical scavenging properties. In addition to lithium lactate, this study also used propylene glycol and methyl paraben as radical scavengers: both materials have good compatibility with the dosimeter without causing excessive optical effects or reacting with the tetrazolium salt [11,18]. Third, this study involved a much more comprehensive set of irradiation conditions: including experiments in small (4 mL), medium (1 L) and large (2.7 L) volumes in order to refine the formulation in clinically relevant volumes. Finally, a simulated single-isocenter multi-target treatment (as a typical use case for the dosimeter) was applied to the optimized formulation for testing against the original formulation.

## 2. Results and Discussion

### 2.1. Multifactorial Design of Experiment

**Figure 1:**
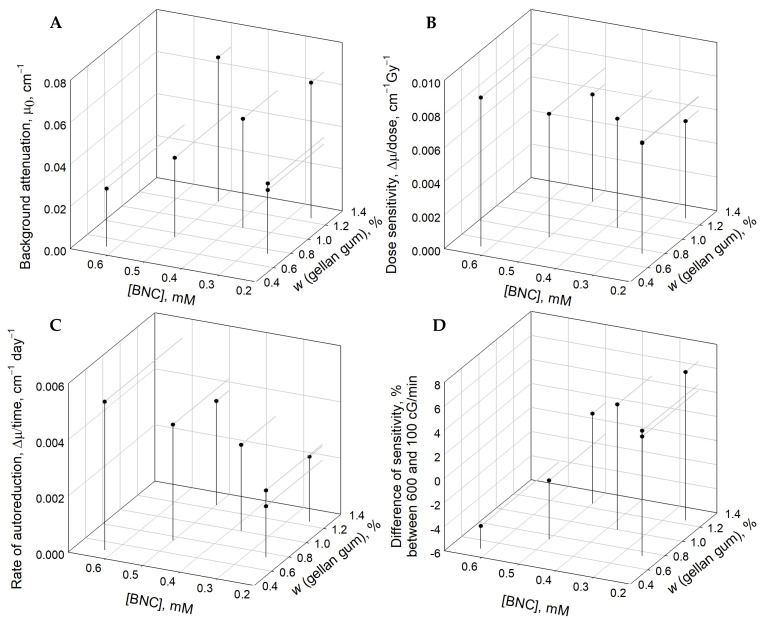
Combined effects of the tetrazolium salt (BNC) and gellan gum concentrations on the dosimetric properties of the gels. **Panel A**: background optical attenuation at 525 ± 5 nm; **panel B**: dose sensitivity, expressed as the change of attenuation per absorbed dose; **panel C**: rate of darkening of the gel while stored at room temperature, expressed as the rate of auto-reduction of the tetrazolium salt, measured as the change of the optical attenuation at 525 ± 5 nm vs. time; **panel D**: dose rate effect, expressed as the percentile difference in dose sensitivity when the gels were irradiated, using 6MV X-ray, at 600 or 100 cGy/min.

The DOE is described in the Materials and Methods section. Figure 1 shows the effects of varying the concentrations of the two main components (gellan gum and the tetrazolium salt) on the experimentally determined dosimetric properties of the gels. As seen in panel A, increasing the gellan gum concentration increased the optical background, due to higher scatter, while the tetrazolium salt (BNC) concentration had a negligible effect on this property. Regarding the dose sensitivity (panel B), both main ingredients had low effects, consistent with prior findings [11], which demonstrated that at BNC concentrations above 0.2 mM, the dose sensitivity approaches a plateau. By contrast, higher BNC concentration led to a higher rate of auto-reduction (panel C) and a decreased dose rate effect (panel D). The gellan gum concentration had a negligible effect on the rate of auto-reduction (panel C), but it impacted the dose rate sensitivity significantly (panel D), where more gellan gum caused a higher difference between the dose sensitivity at high and low dose rates (panel D). These results indicate that gellan gum is involved in the radiochemical process but not in the auto-reduction reaction, where the radiation-sensitive tetrazolium salt is reduced into formazan during storage.

**Figure 2:**
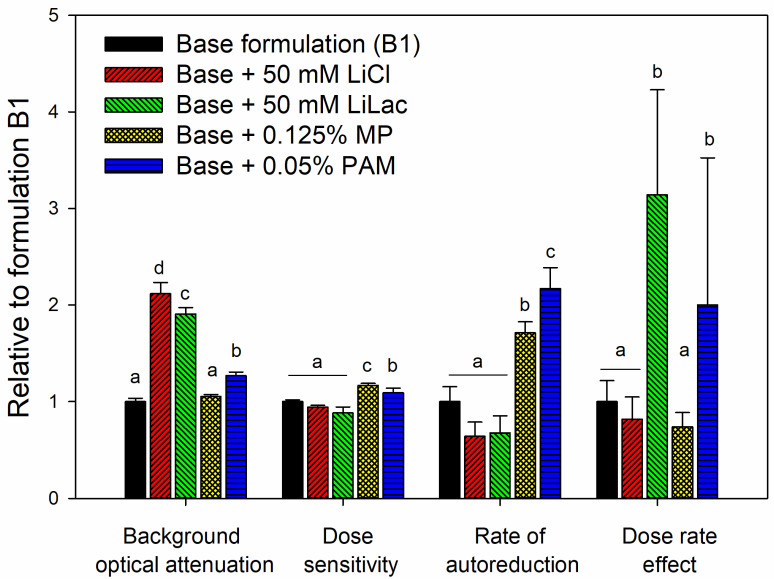
Effects of individual ingredients on the properties of the dosimeter: background optical attenuation, dose sensitivity, rate of auto-reduction and dose rate effect, as defined before, normalized to the control (formulation B1: 0.75% gellan gum and 0.25 mM BNC). Significantly different values (*p* < 0.05) in each category are shown by different letters from “a” to “d” in order of increasing magnitude. For example, the background attenuation was not significantly affected by the addition of methyl paraben (MP), while the addition of polyacrylamide (PAM), lithium lactate (LiLac) or lithium chloride (LiCl) increased the attenuation in the same order. It should be noted that the dose rate effect, which is a difference of sensitivities, is strongly affected by experimental uncertainties in small-scale samples, hence the large error bars.

Figure 2 shows the effects of all ingredients, other than BNC and gellan gum, on the dosimetric properties of the gel. The results were normalized to the properties of the formulation containing 0.75% gellan gum and 0.25 mM BNC without other additives. The crosslinking agents (either lithium chloride or lithium lactate) increased the optical background, likely due to higher optical scattering. However, they were required at that low gellan gum concentration in order to maintain the mechanical strength of the gel [18,23]. The buffered lithium lactate did not significantly affect the sensitivity of the dosimeter, but it did cause a much higher dose rate effect; therefore, this additive was excluded in further experimentations. The addition of either methyl paraben or polyacrylamide improved the sensitivity at the cost of faster auto-reduction, and in the case of PAM, a larger dose rate effect. However, the last two materials have the added benefit of providing antifouling protection and lower syneresis, respectively [18], so they were deemed essential.

Rather than examining the multiple analysis of variance for each experimental set, the least square approach was chosen in order to arrive at an explanatory model for the properties of the dosimeters. The individual effects of the tested additives were modelled using a linear equation based on a least squares regression on all available data points from the formulations in sets A to C. The linear equation indices were each tested against the null hypothesis that they have no explanatory power and rejected from the model if that probability (*p*) was higher than 5%. The overall model equation is:

(1) $$f_{i}=a_{i,0}+a_{i,1}\omega_{\mathrm{Gn}}+a_{i,2}C_{\mathrm{BCN}}+a_{i,3}C_{\mathrm{LiCl}}+a_{i,4}C_{\mathrm{LiLac}}+a_{i,5}\omega_{\mathrm{MP}}+a_{i,6}\omega_{\mathrm{PAM}}$$

where the function *f_i_* represents the individual dosimeter property *i*, *a_i,j_* are the equation indices (*j* = 0 to 6), *ω* is the weight fraction (in %), and *C* is the concentration (in M or mM) of the individual components, as specified in Table 1. From Table 1, within the studied experimental space, the linear model showed a good fit with *R*^2^ values in decreasing order of 0.98 for the background attenuation, 0.95 for the rate of auto-reduction, 0.94 for the dose sensitivity, and 0.90 for the dose rate effect, respectively. The same general trends seen in Figure 1 and Figure 2 were observed. First, the background optical attenuation increased with increasing the concentrations of each of the ingredients except MP. Second, the dose sensitivity increased as the BNC and MP concentrations increased but dropped at higher gellan gum concentrations and was negatively affected by the presence of buffered lithium lactate. Third, the rate of auto-reduction increased as the BNC, MP, and PAM concentrations increased, while it decreased in the presence of either of the lithium salts. Forth, the dose rate effect either increased or was unaffected as the concentrations of most components rose, dropping only by increasing the BNC concentration.

The model (Equation (1)) can be used to predict the properties of new gel dosimeter formulations. Thus, formulations D1, D2, and D3 (Table 2) were selected so the dose rate effect between 100 and 600 cGy/min would be under 2%.

### 2.2. Depth-Dose Curves

**Figure 3:**
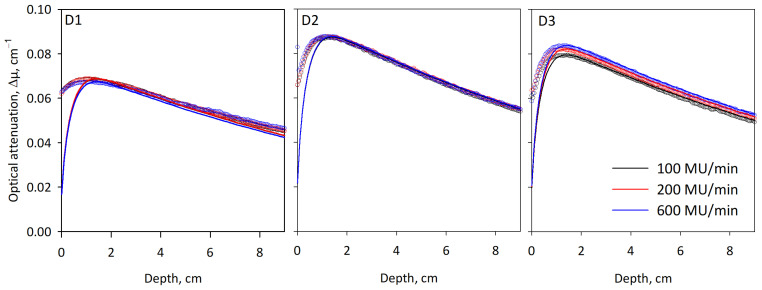
Depth dose curves in 1-L volumes of formulations D1 to D3 using 6MV X-rays (2 × 2 cm^2^ field) as measured (open symbols) compared to the Monte-Carlo calculations (solid lines). The same y-axis scale was used in all cases to demonstrate the difference in sensitivity. Over the whole dose-depth curve, there was a relatively weak dependence between the measured and calculated results (*R*^2^ < 0.79), with a marked over-response in the dose build-up region, possibly due to the effect of dissolved oxygen near the surface of the gel. Below the depth of maximum dose (*d*_max_ = 1.6 cm), *R*^2^ reached above 0.998 for all cases.

Figure 3 presents the depth dose curves obtained in formulations D1–D3 by 6MV X-ray square beams (2 × 2 cm^2^) delivered off-axis to 1-L volumes of the gels at three different dose rates (100, 200 and 600 cGy/min). The dose rate effect between 100 and 200 cGy/min increased from less than 0.5% in formulation D1 to 1% in D2 and 2% in D3, but formulation D2 had the most consistent behavior at all three dose rates. Whereas the depth-dose curves were well-fit with the Monte-Carlo calculation below the depth of maximum dose (*d*_max_), there was a noticeable over-response in the dose build-up region close to the top of the gel, which may be attributed to the effect of the dissolved oxygen near the surface of the gel. A similar effect was previously reported in fresh samples of ClearView [24] but was absent in well-sealed and aged gels [16,19]. To avoid uncertain dose sensitivity near the top surface of the gel, the jars used in the follow-up tests were sealed with heat-adhesive aluminum foil and aged for a week before use.

In Table 2, the model predictions are presented against the experimental findings for two crucial dosimeter characteristics—the dose sensitivity (as determined at a dose rate of 600 cGy/min) and the dose rate effect. The predicted and experimental results in both categories followed the same trends, as evidenced by the high correlation coefficients (*R*^2^ > 0.96), but the experimental data for the dose rate effect showed a much wider spread than expected. Of the three formulations, only D2 remained in the limit of 0–2%, according to which these formulations were derived.

### 2.3. Simulated Single Isocenter Multitarget Treatment

**Figure 4:**
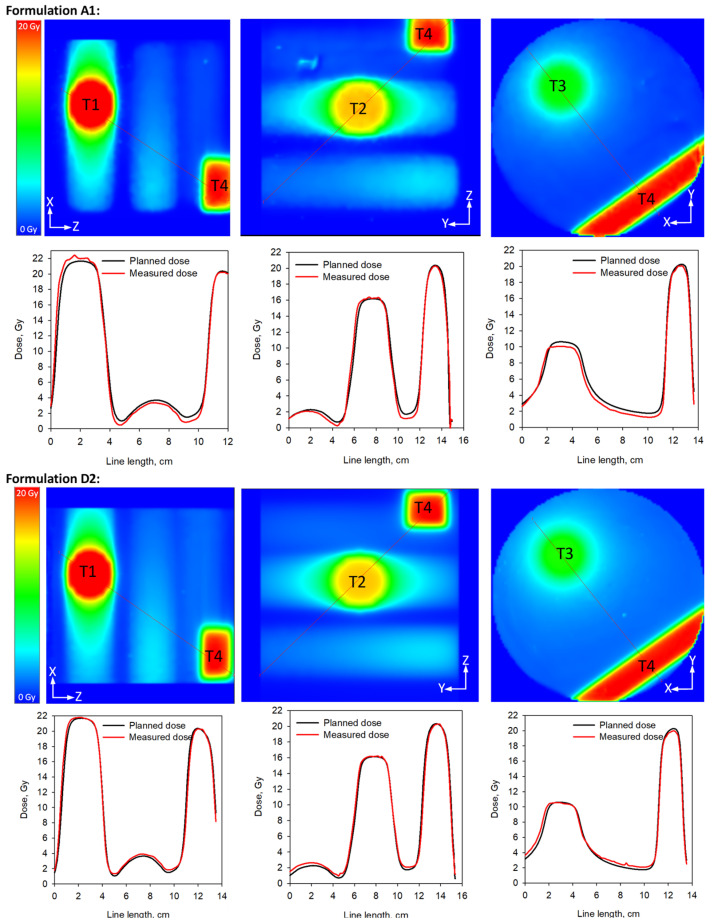
Line profiles in gels from formulations A1 (original ClearView) and D2 (reformulated ClearView) through three spherical targets (T1–T3) with 30 mm diameter each and a calibration target T4 made from two square parallel-opposed beams of 2 × 2 cm^2^. The nominal doses and dose rates for each target were: T1: 20 Gy at 600 cGy/min, T2: 15 Gy at 300 cGy/min, T3: 10 Gy at 100 cGy/min, and T4: 20 Gy at 400 cGy/min.

To confirm the results from the scale-up testing and to demonstrate the effect of the optimization process, formulations A1 (original ClearView) and D2 were compared directly by irradiation in large volume samples (2.7 L) using a single isocenter multi-target delivery to three spherical targets and a calibration volume, as detailed in the Materials and Methods section. Figure 4 presents line profiles through each of the targets. Noticeably formulation A1 showed an over-response in the high-dose (high dose rate) regions (T1), and an under-response in the low-dose (low dose rate) regions (T3). This effect was mostly absent in the response of D2. A slight geometric shift (<2 mm) is seen in the XY plane in both compositions, which may be due to optical effects in the scanner.

To further evaluate the performance of the two formulations, for each case, the calculated and measured dose distributions were compared by calculating the γ index for dose-difference and distance-to-agreement criteria not higher than 3%/2 mm, as recommended by the American Association of Physicists in Medicine (AAPM) task group No. 218 [25]. To use the γ test for the volumes of the gels, the resolution of the measured data was resampled to match the resolution of the treatment plan, then the displacement (Γ) between each evaluated and reference point (${\vec{r}}_{e}$ and ${\vec{r}}_{r}$, respectively) was determined according to [26]:

(2) $$\Gamma \left({\vec{r}}_{e},{\vec{r}}_{r}\right)=\sqrt{\frac{r^{2}\left({\vec{r}}_{e},{\vec{r}}_{r}\right)}{{\left(\Delta d\right)}^{2}}+\frac{\delta^{2}\left({\vec{r}}_{e},{\vec{r}}_{r}\right)}{{\left(\Delta D\right)}^{2}}}$$

where $r\left({\vec{r}}_{e},{\vec{r}}_{r}\right)$ and $\delta \left({\vec{r}}_{e},{\vec{r}}_{r}\right)$ are the distance-to-agreement (mm) and dose difference (%), while Δ*d* and Δ*D* are the respective criteria. Then the γ function for each reference point (${\vec{r}}_{r}$) is the minimum displacement for all evaluated points:

(3) $$\gamma \left({\vec{r}}_{r}\right)=\min\left\{\Gamma \left({\vec{r}}_{e},{\vec{r}}_{r}\right)\right\},\forall {\vec{r}}_{e}$$

For a specific reference point to pass, $\gamma \left({\vec{r}}_{r}\right)$ should not be higher than 1. This means that there exists at least one evaluated point within the sphere with a radius Δ*d* mm around the reference point where the measured dose is within Δ*D*% from the reference dose. For a treatment plan to pass, the gamma passing rate (GPR), defined as the fraction of points for which γ ≤ 1 over the whole volume, should not be lower than 95%. Table 3 shows the percent GPR at criteria ranging from the very strict 1%/1 mm to the recommended 3%/2 mm. Considering that the irradiations were performed using the same treatment plan on the same machine in a single session, the difference between the GPRs should depend primarily on the gel formulations.

**Figure 5:**
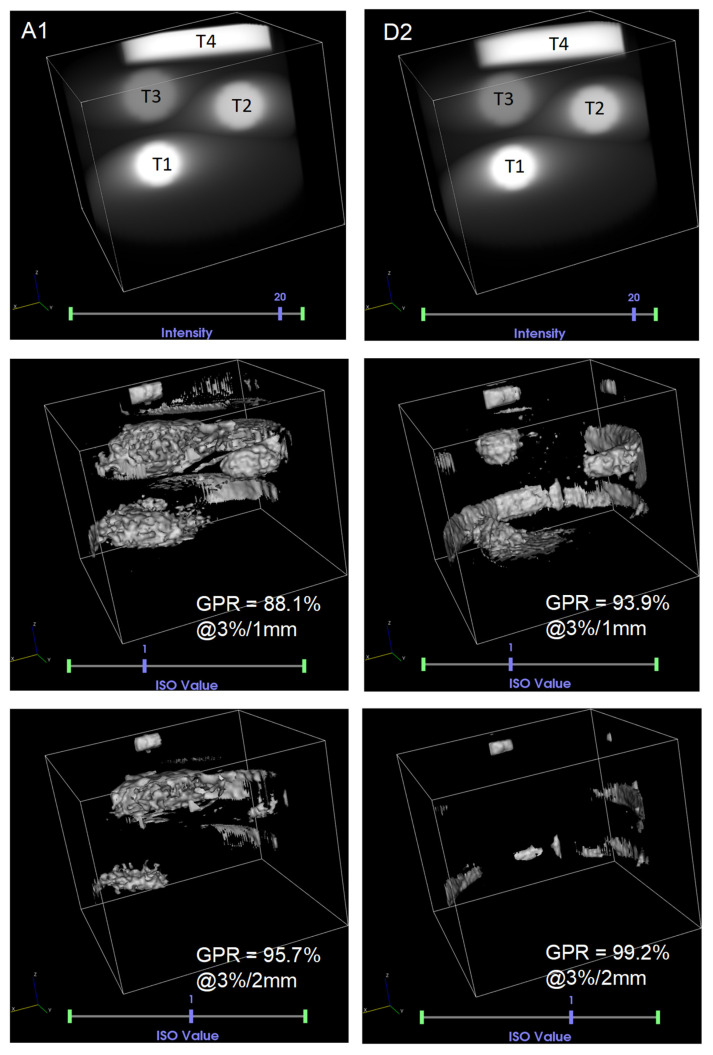
3D maps of the dose treatment plan in cylindrical volumes (diameter 15 cm, height 12 cm), normalized to 20 Gy for formulations A1 and D2 (top). The nominal doses and dose rates for each target were: T1: 20 Gy at 600 cGy/min, T2: 15 Gy at 300 cGy/min, T3: 10 Gy at 100 cGy/min, and T4: 20 Gy at 400 cGy/min. Compared to isometric maps at γ > 1 (failing points) at percent dose-difference and distance to agreement of 3%/1 mm (middle) and 3%/2 mm (bottom) at 10% minimum dose threshold in the same volumes. Notably, while formulation A1 passes with 95.7% GPR at 3%/2 mm, low dose rate regions failed, including the whole volume T3, as well the build-up regions of T2 and T1; whereas, at the same criteria, formulation D2 passed everywhere except near the periphery of the volume.

Neither of the formulations passed the strict 1 mm distance-to-agreement criterion within a 3% dose difference. That may be partially due to uncertainties in the treatment planning and delivery, as well as the gel chemistry and optical effects in the scanner. At 2 mm distance-to-agreement, formulation D2 passed with higher GPR and in more stringent conditions (2%/2 mm), while formulation A1 passed only at 3%/2 mm. To further examine these effects, Figure 5 shows how the two formulations passed (or failed) under select γ test criteria.

From Figure 5, it appears that at 3%/2 mm, formulation A1 fails in the low-dose (and low-dose rate) region even if the overall GPR is over 95%. By comparison, while formulation D2 also fails in the low dose regions at 3%/1 mm, it passes everywhere except near the jar walls at the 3%/2 mm criterion. This result suggests that there is a measurable and clinically relevant improvement in the optimized gel formulation.

## 3. Conclusions

Radiochromic gel dosimeters provide an appealing approach to the three-dimensional quality assurance of radiation therapy due to their favorable radiological properties, fast response to radiation, and inexpensive optical readout. In particular, the ClearView 3D dosimeter, which is based on a tetrazolium salt dispersed in a gellan gum gel, additionally provides extended physical and chemical stability of the gel and lack of diffusion of the radiation image, thus, improving the handling and logistics of 3D dosimetry. However, the previously released ClearView formulation presented a marked dose rate effect which limited its application in clinical settings. In this paper, a reformulation of the dosimeter was presented based on a design of experiments approach, starting with small-volume tests (in 4-mL cuvettes), confirming the results in larger volumes (1-L jars) and testing the final formulation in clinically relevant volumes (2.7 L jars) using a complex treatment delivery.

Relative to the starting formulation A1 (original ClearView) which contained 1.25% gellan gum and 0.25 mM BNC tetrazolium, the final optimized formulation (D2) contained lower amount of gellan gum (0.75%), and slightly elevated BNC concentration (0.375 mM), with the addition of 50 mM LiCl (crosslinker), 0.125% MP (radical scavenger) and 0.05% PAM (rheology modifier). The optimized formulation D2 outperformed A1 when treated to a complex radiation delivery by passing the 3%/2 mm percent dose-difference and distance to agreement criterion in 99.2% of the treated volume, even in the most challenging low dose and low-dose rate regions, compared to 95.7% for formulation A1 which failed in some low-dose rate regions. This result may find clinical application in the quality assurance of highly complex treatment plants with dynamic dose and dose rate prescriptions.

## 4. Materials and Methods

### 4.1. Materials and Design of Experiments

Gellan gum (Gn), methyl paraben (MP), propylene glycol (PG), lithium chloride (LiCl), lithium lactate (LiLac), lactic acid and poly(acrylamide) with a molecular weight of 5 to 6 MDa (PAM) were purchased from Fisher Scientific (Ottawa, ON, Canada) and 2,3-bis(4-nitrophenyl)-5-phenyltetrazolium chloride (BNC) from TCI America (Portland, OR, USA). All formulations contained 10% PG as a radical scavenger, while the concentrations of the other components were optimized in a multifactorial design of experiment (DOE) (Table 4). In set A, a two-level (low and high), two-factor DOE was implemented to test the effects of the two main components (gellan gum and BNC). The central point (formulation A5) was added to evaluate the linearity of the model, and formulation A6 was added to demonstrate the extrapolation of the model close to the DOE space. In set B, the individual effects of the additives were tested at a single concentration for each of them, against a base formulation (B1, equivalent to A3). The concentration of the lithium salts was based on prior studies of crosslinking of gellan gum [18]. In the case of lithium lactate, which is slightly alkaline, dilute lactic acid was used to adjust the pH of the solution to 5.3. Methyl paraben was added at approximately 85% of its solubility in water to avoid precipitation, and the amount of PAM was chosen based on preliminary tests of the handling of the gels. In set C, select interactions between the additives were examined while maintaining the total polymer loading constant at 0.75%, to allow direct comparison against the base formulation B1. It should be noted that not all formulations in sets A to C are suitable for scale-up, as the gels may be too weak. By contrast, the formulations in set D are all suitable for scaling up for further testing under clinically relevant conditions.

### 4.2. Experimental Set-Up

The formulations in sets A to C were dispersed into 4-mL polymethylmethacrylate cuvettes and irradiated in triplicates under 5-cm of water with a 6 MV flat X-ray field (10 × 10 cm) at 100 and 600 cGy/min on a Clincac iX linear irradiator by Varian (Palo Alto, CA, USA). Optical readout was performed at 525 ± 5 nm before and after irradiation and during aging at room temperature for 14 days. The formulations in set D were prepared in 1-L clear polyethylene terephthalate jars (useful dimensions 10 cm diameter, 12 cm height), and irradiated from the top with three square X-ray fields (2 × 2 cm^2^) at 100, 200 and 600 cGy/min, at a source-to-surface distance of 95.0 cm, to a dose of 12 Gy at 5.0 cm below the gel surface. The jars were scanned optically before and after irradiation on the Vista 16 convergent light source optical computed tomography scanner from Modus QA (London, ON, Canada), and percentage depth-dose (PDD) curves were generated against in-house Monte-Carlo calculations based on the EGSnrc code. Selected formulations were also prepared in larger 2.7-L polyethylene terephthalate jars (useful dimensions 15 cm diameter, 12 cm height) that, following scanning on a Large Bore CT scanner by Philips (Amsterdam, Netherlands) and treatment planning on Eclipse v15.6 (Varian), were irradiated with 6 MV X-ray on a TrueBeam linear irradiator (Varian). Three 3.0-cm spherical planning target volumes (PTVs) were contoured in positions off the central axis to simulate a multiple-lesion brain stereotactic radiosurgery treatment. The spherical PTVs were all planned with an MLC-defined conformal arc technique to maintain a stable dose rate in each case: 10 Gy at 100 cGy/min, 15 Gy at 300 cGy/min, and 20 Gy at 600 cGy/min. An additional calibration plan of a two-field parallel opposing fields to a mean dose of 20 Gy at 400 cGy/min was contoured to a 2 × 2 cm^2^ cross-section. Before and after irradiation, the gels were scanned on the Vista 16 scanner, and image analysis was done with Vista ACE processing software (Modus QA).

## Figures and Tables

**Table 1:** Linear effects (as defined in Equation (1)) with associated probabilities for the null hypothesis (*p*). For statistical significance, *p* < 0.05. *R*^2^ is the coefficient of determination, showing the explanatory power of each model equation against the measured dosimeter properties.

Factor	Base Unit	Background, 10^3^ × μ_0_, cm^−1^		Dose Sensitivity 10^3^ × *S*, cm^−1^ Gy^−1^		Auto-Reduction 10^3^ × *R*, cm^−1^ day^−1^		Dose Rate Effect, % at 600 vs. 100 cGy/min	
		Coeff.	*p*	Coeff.	*p*	Coeff.	*p*	Coeff.	*p*
Constant		−20.4 ± 3.4	0.004	7.38 ± 0.46	<0.001	n.s.	0.381	5.64 ± 1.62	0.025
Gn	%	61.8 ± 2.5	<0.001	−2.25 ± 0.34	0.003	n.s.	0.454	4.72 ± 1.21	0.017
BNC	mM	25.6 ± 4.7	0.005	3.76 ± 0.64	0.004	7.60 ± 0.82	<0.001	−19.5 ± 2.3	<0.001
LiCl	M	634 ± 39	<0.001	n.s.	0.177	−20.8 ± 6.9	0.039	n.s.	0.494
LiLac	M	506 ± 39	<0.001	−16.7 ± 5.4	0.036	−19.7 ± 6.9	0.046	189 ± 19	<0.001
MP	%	n.s.	0.805	8.32 ± 2.14	0.018	7.15 ± 2.76	0.06	n.s.	0.325
PAM	%	182 ± 40	0.011	n.s.	0.195	34.2 ± 7.0	0.008	94.5 ± 19.3	0.008
*R* ^2^		0.989		0.938		0.949		0.896	

n.s. = not significant.

**Table 2:** Results from the percentage depth-dose curves below *d*_max_ in 1-L jars against the model (Equation (1), Table 1) derived from cuvette experiments for the dose sensitivity in cm^−1^Gy^−1^ at a dose rate of 600 cGy/min and the dose rate effect, expressed as the difference in dose sensitivity between 600 and 100 cGy/min.

	Ingredients					Dose Sensitivity 10^3^ × *S*, cm^−1^Gy^−1^		Dose Rate Effect, % at 600 vs. 100 cGy/min	
	ω_Gn_ %	*C*_BNC_ mM	*C*_LiCl_ M	ω_MP_ %	ω_PAM_ %	Model	Experiment	Model	Experiment
D1	1.25	0.57	0	0	0	6.82	6.23 ± 0.09	0.42	−1.73 ± 0.12
D2	0.70	0.375	0.05	0.125	0.05	8.59	8.76 ± 0.07	1.23	0.61 ± 0.10
D3	0.60	0.30	0.10	0.125	0.05	8.15	8.12 ± 0.07	1.98	4.92 ± 0.10
*R* ^2^							>0.999		0.964

**Table 3:** Gamma passing rates (GPR) at 10% minimum dose threshold, comparing the dose difference (%) and distance to agreement (mm) between the treatment planning system and the measured dose. Failing GPR are bracketed; passing GPR are in bold; 95% of the tested volume at γ ≤ 1 is required to pass.

	1%/1 mm	2%/1 mm	3%/1 mm	1%/2 mm	2%/2 mm	3%/2 mm
A1	(56.4%)	(75.2%)	(88.1%)	(77.7%)	(88.7%)	**95.7%**
D2	(39.3%)	(81.1%)	(93.9%)	(66.9%)	**96.6%**	**99.2%**

**Table 4:** All tested formulations contained 10% propylene glycol, gellan gum (Gn) and BNC tetrazolium salts. The tested additives were lithium crosslinkers (LiX), lithium chloride (LiCl) or buffered lithium lactate (LiLac), the radical scavenger methyl paraben (MP) and the rheology modifier polyacrylamide (5–6 MDa) (PAM).

Formulation	ω_Gn_ %	*C*_BNC_ mM	*C*_LiX_ mM	ω_MP_ %	ω_PAM_ %
Set A. Two-factorial study of Gn and BNC					
A1	1.25	0.25	–	–	–
A2	1.25	0.50	–	–	–
A3	0.75	0.25	–	–	–
A4	0.75	0.50	–	–	–
A5	1.00	0.375	–	–	–
A6	0.50	0.625	–	–	–
Set B. Individual effects of additives					
B1	0.75	0.25	–	–	–
B2	0.75	0.25	50 LiCl	–	–
B3	0.75	0.25	50 LiLac	–	–
B4	0.75	0.25	–	0.125	–
B5	0.75	0.25	–	–	0.05
Set C. Combined effects of additives					
C1	0.70	0.25	–	–	0.05
C2	0.70	0.25	50 LiCl	–	0.05
C3	0.70	0.25	–	0.125	0.05
C4	0.70	0.25	50 LiLac	0.125	0.05
C5	0.70	0.375	50 LiLac	0.125	0.05
C6	0.70	0.50	50 LiLac	0.125	0.05
Set. D. Model-derived formulation					
D1	1.25	0.57	0	0	0
D2	0.75	0.375	50 LiCl	0.125	0.05
D3	0.60	0.30	100 LiCl	0.125	0.05

## Data Availability

Data is available upon request.
